# Comparison of global indicators for severe maternal morbidity among South Korean women who delivered from 2003 to 2018: a population-based retrospective cohort study

**DOI:** 10.1186/s12978-022-01482-y

**Published:** 2022-08-13

**Authors:** Jin Young Nam

**Affiliations:** Department of Healthcare Management, Eulji University, Sanseongdae-ro 553, Sujeong-gu, Seongnam, Gyeonggi-do 13135 Republic of Korea

**Keywords:** Severe maternal morbidity, International SMM indicators, Risk factors, Adverse obstetric factors

## Abstract

**Background:**

Even though several severe maternal morbidity (SMM) indicators exist globally, indicators that can serve as international standards are needed. Therefore, this study aimed to compare the SMM risk assessment using four international indicators and identify the factors underlying the differences among the risk assessments obtained by the various indicators.

**Methods:**

This study used the National Health Insurance delivery cohort in South Korea from 2003 to 2018. SMM was estimated using four indicators: the United States Centers for Disease Control and Prevention (US-CDC) SMM algorithm, the American College of Obstetricians and Gynecologists (ACOG) gold standard guidelines, Zwart et al.’s indicators for the Netherlands, and the European Network on Severe Acute Maternal Morbidity (EURONET-SAMM) index. Generalized estimating equations models were used to identify the relationships between SMM indicators and risk factors.

**Results:**

The SMM incidence rates in 6,421,091 deliveries, were 2.36%, 3.12%, 0.31%, and 1.36% using the US-CDC, ACOG, Zwart et al.’s, and EURONET SAMM indicators, respectively. In sub indicators, hemorrhage-related codes constituted the highest proportion of all SMM indicators. Advanced maternal age was related to high risk in all four SMM indicators (US-CDC: 40–44 years, RR 1.67, 95% CI 1.63–1.71; ACOG’s guidelines: 40–44 years, RR 1.52, 95% CI 1.49–1.56; Zwart’s indicators: RR 2.72, 95% CI 2.55–2.90; EURONET-SAMM: RR 2.04, 95% CI 1.97–2.11) compared to those aged 25–29 years. In residential area, women who lived in rural area had approximately 1.2- to 1.5-fold higher risk of SMM compared to those who lived in Seoul. Additionally, inadequate prenatal care was associated with a 1.1- to 1.4-fold higher risk of SMM compared to adequate prenatal care.

**Conclusions:**

SMM was associated with maternal age, socioeconomic status, and adverse obstetric factors using various international SMM indicators. Further studies are needed to further determine risk and preventable factors for SMM and to identify more specific causes associated with the frequent sub-indicators of SMM.

**Supplementary Information:**

The online version contains supplementary material available at 10.1186/s12978-022-01482-y.

## Plain language summary

There are several indicators of severe maternal morbidity (SMM) globally, but indicators that can serve as international standards are not exist yet. This study compared the SMM risk assessment using four international indicators such as US-CDC’s SMM, ACOG’s gold standard guidelines, Zwart et al.’s SMM, and EURONET-SAMM, and identify the factors underlying the differences among the risk assessments obtained by the various indicators.

This study extracted women who were aged 15–49 years, those who had childbirth in the healthcare institute during 2003 to 2018 in South Korea using the National Health Insurance database.

Of the 6,421,091 childbirth cases, the incidence of each SMM indicators were as follow: the US-CDC’s SMM: 2.4%; the ACOG’s gold standard guidelines: 3.1%; Zwart et al.’s SMM: 0.3%; the EURONET-SAMM: 1.4% indicators. In addition, the highest incidence of each sub-indicators was blood transfusion or obstetric hemorrhage which recorded more than 70% of total SMM cases. In particular, the risk factor on SMM were: advanced maternal age; living rural area; inadequate prenatal care.

In conclusion, SMM was associated with maternal age, socioeconomic status, and adverse obstetric factors using various global SMM indicators. Therefore, further studies are needed to identify more specific causes associated with the frequent sub-indicators of SMM and to determine risk and preventable factors for SMM.

**Supplementary Information:**

The online version contains supplementary material available at 10.1186/s12978-022-01482-y.

## Background

The fifth goal of the United Nations Millennium Development Goals is to reduce the global maternal mortality ratio (MMR), which is also the first indicator of the health-related Sustainable Development Goals. Efforts to reduce the global MMR and promote the improvement of maternal health are ongoing worldwide [1, 2]. However, since delivery-related maternal mortality is a relatively rare event and given the difficulty in predicting in which women it will occur in, conducting research in maternal mortality prevention is particularly challenging because of the sparsity of events and inadequate relevant information [3, 4]. Thus, the development of proxy indicators is needed.

In 2011, the World Health Organization (WHO) identified severe maternal morbidity (SMM) indicators specifically associated with maternal death [5, 6]. Such real-time measurements could be taken during childbirth but this is often not possible in developing countries or in rural areas of developed countries lacking human resources and measurement infrastructure [5, 6]. In 2012, the United States Centers for Disease Control and Prevention (US-CDC) published a list of 21 SMM indicators, including the corresponding diagnostic and procedure codes. As these can be measured using administrative data, and is not reliant on real-time measurement, US-CDC SMM indicators are easier to measure than those proposed by WHO. However, given that the assessment is based on the presence or absence of a related diagnosis and procedure, the severity of maternal morbidity cannot be measured [7]. More recently, the American College of Obstetricians and Gynecologists (ACOG) announced new gold standard guidelines for SMM indicators, which include the length of childbirth hospitalization, intensive care unit admission, blood transfusion, and readmission within 30 days of delivery, in addition to the US-CDC SMM indicators [8]. Additionally, other indicators were developed in Europe; Zwart et al.’s indicators for the Netherlands, [9] and the European Network Group on Severe Acute Maternal Morbidity (EURONET-SAMM) indicators which were developed by researchers and public health professionals from eight European countries. The SAMM indicators selected seven principles and confirmed the classification of diagnostic and procedure codes for SAMM [10]. Even though several SMM indicators exist globally, selecting indicators that can serve as an international standard remains controversial.

In South Korea the risk of SMM is increasing, in the face of a rather high MMR among OECD countries, which was recorded at 11.8 per 100,000 live births, [11] paralleled by an increasing trend for high-risk pregnancies, such as advanced-age pregnancies [12] and multiple pregnancies [13]. However, there is limited related research, and there are few official proxy indicators of MMR. Against this background, this study aimed to compare SMM incidence in South Korean women using four different international SMM indicators, i.e., those of the US-CDC, the ACOG, Zwart et al., and the European Network on Severe Acute Maternal Morbidity [EURONET]), as proxy indicators of MMR. This study aimed to compare the SMM risk assessment by the use of four international indicators and identify the factors underlying the differences among the risk assessments obtained by the various indicators. This is the first study to compare the international indicators for SMM in the population who delivered in South Korea over the 16-year period.

## Methods

### Data source and population

In this population-based cohort study, data were collected on all women of childbearing age (15–49 years) who had delivered children at medical facilities from 2003 to 2018, using the National Health Insurance (NHI) database, which consists of health-care utilization data, physical checkup data, sociodemographic data, and mortality data. The NHI, as the only health insurer in South Korea, stores cohort data collected during the claims process and includes records of hospitalizations, outpatient care, and drug prescriptions. This database also stores information related to health-care utilization, such as age, sex, residential area, insurance type, income, diagnostic codes, procedure codes, prescription drugs, individual medical expenses, and information on the hospitals covered by the NHI. The NHI cohort data can be used to continuously track the characteristics of patients, clinical records, and health-care providers; indicate the epidemiologic causes of disease; and provide information on the development of health-care policies. These data are anonymized by assigning unique number codes so that personal patient information remains unidentifiable [14]. The study was conducted according to the guidelines of the Declaration of Helsinki and approved by the Institutional Review Board (SMWU-1808-HR-076) of Sookmyung Women’s University.

An analysis period of at least 310 days (280 days of full-term pregnancy + 30 postpartum days) is necessary to check for maternal comorbidities during pregnancy and health status during the puerperal period; therefore, only women who had delivered at hospitals during the period from January 1, 2003 to December 1, 2018 and who had hospitalization records were defined as the study population. Postpartum women were defined as women who delivered at a hospital and who had hospitalization records that included an electronic data interchange code consisting of a diagnostic code; recorded as a single vaginal delivery (O80), a single delivery with forceps and vacuum extraction (O81), a single delivery by cesarean section (O82), a single delivery by any other supportive device (O83), or multiple births (O84); and a procedural code, recorded as normal delivery, breech extraction, cesarean section, or forceps or vacuum extraction. In total, 6,421,091 mothers who had delivered children from 2003 to 2018 were included in the study analysis.

### SMM indicators

With SMM as the dependent variable, the analysis was performed using the following SMM indicators: (1) the SMM algorithm, defined by the US-CDC; (2) the new gold standard guideline for SMM, defined by the ACOG; (3) Zwart et al.’s. established SMM criteria in the Netherlands; and (4) the EURONET-severe acute maternal morbidity (EURONET-SAMM) index in eight European countries.

The US-CDC’s SMM algorithm defined SMM as the occurrence of at least one of a possible total of 21 indicators, consisting of 16 diagnostic codes and six procedural codes during the postnatal hospital stay [7, 15].

SMM criteria, as defined by the ACOG, are as follows: (1) the occurrence of at least one of 21 SMM indicators, as defined in the US-CDC’s SMM algorithm; (2) prolonged length of postnatal hospital stay; (3) intensive care unit (ICU) admission; (4) transfusion of ≥ 4 units of packed red blood cells; and (5) hospital readmission within 30 days of discharge [8]. In this study, all ACOG’s gold standard guidelines were adopted for analysis except the indicator on length of postnatal hospital stay because of the different criteria for postnatal hospital stays in the US and South Korean medical delivery systems. In the US, postnatal hospital lengths of stay > 3 days and ≥ 6 days are classified as SMM events for vaginal delivery and cesarean section, respectively, while the median length of postnatal hospital stays in South Korea is 3–4 days for vaginal delivery and 6–7 days for cesarean section. Therefore, application of the US standard to Korean cases would classify > 50% of all deliveries as SMM cases. Applying US standards to Korean women might be overestimated; accordingly, among the ACOG criteria, the length of hospital stay was excluded from the scope of analysis of this study.

In the Netherlands, Zwart et al. established SMM criteria, in agreement with the Dutch Maternal Mortality Committee of the Dutch Society of Obstetrics and Gynaecology, as the occurrence of one or more of the following postpartum events: (1) admission to the ICU, (2) uterine rupture, (3) eclampsia, (4) transfusion of ≥ 4 units of packed red blood cells, and (5) other SMM, according to the opinion of the treating obstetrician [9].

Another European standard is the EURONET-severe acute maternal morbidity (EURONET-SAMM) index, established through extracting and comparing the SAMM data of eight European countries, namely, Finland, France, Italy, Portugal, Switzerland, England, Scotland, and Wales, using their respective national hospital discharge records and determining the final codes, which consisted of diagnostic and procedural codes. Patients with any one of five SAMM indicators (eclampsia, septicemia during pregnancy, pregnancy-related hysterectomy, hysterectomy associated with a diagnosis of obstetric hemorrhage, and red blood cell transfusion associated with a diagnosis of obstetric hemorrhage) are classified as having SAMM [10].

Of the international indicators identified through this literature review, those recognizable as claims data were preselected as diagnostic and procedural codes, and the final SMM codes were established with the involvement of obstetrician-gynecologist, medical record administrators, and data scientists.

### Covariates

Personal, obstetric, and provider factors were set as covariates. Personal factors included maternal age (range: 15–19, 20–24, 25–29, 30–34, 35–39, 40–44, and ≥ 45 years), household income (divided into quartiles: Q1 [low]), Q2, Q3, and Q4 [high]), type of health insurance (coverage according to region for those who were self-employed; coverage according to workplace for employees; and medical aid), and residential area (Seoul, metropolitan cities, small cities, and rural areas). Obstetric factors included mode of delivery (vaginal delivery, instrumental delivery, and caesarean section), preterm births [No (≥ 37 weeks’ gestation) and Yes (< 37 weeks’ gestation)], parity (primiparous and multiparous), and multiple births (single and multiple embryos). Prenatal care was estimated using Kessner’s adequate prenatal care index [16]. Prenatal care was rated adequate when a woman began prenatal care in the first trimester and had nine prenatal care visits for a normal-length pregnancy [16]. Prenatal care was determined inadequate if a woman began prenatal care after the third trimester and had less than four prenatal care visits. All the other situations in between were classified as intermediate prenatal care [16]. Maternal comorbidities were defined according to Howell’s criteria, [17] thus including cardiac disease, renal disease, musculoskeletal disease, digestive disorder, blood disease, mental disorders, CNS disease, rheumatic heart disease, placentation disorder, chronic hypertension, pregnancy hypertension, lupus, collagen vascular disorder, rheumatoid arthritis, diabetes, diabetes complicating pregnancy, obesity, and asthma/chronic bronchitis. Provider factors included the type of hospital according to number of beds (> 500 beds, 100–499 beds, 30–99 beds, and < 30 beds) and hospital location (Seoul, metropolitan city, small city, and county).

### Statistical analysis

Based on the customized datasets obtained from the NHI system, all women who delivered children from 2003 to 2018 were analyzed. Pearson’s Chi-square tests were performed to ascertain differences in sociodemographic characteristics and their distribution between SMM cases and the study population, during labor and delivery hospitalization, based on the established codes. The frequencies and fractions of sub-indicators from each of the four SMM indicators were calculated and compared using basic statistics. Finally, the adjusted relative risk (RR) and 95% confidence intervals (CIs) were calculated using a generalized estimating equations model at a significance level of *P* < 0.05 to estimate the relationships between each SMM indicator and the demographic, obstetric, and provider factors. Data analysis was performed using SAS 9.4 software (SAS Institute, Inc., Cary, NC, USA).

## Results

Table 1 shows the incidence of SMM calculated using the four SMM indicators and the distribution of the study population’s general characteristics (see Additional file 1 for more information on the results of chis-square test). The SMM rates during the analysis period (2003–2018) were 2.36% (n = 151,533) of a total of 6,421,091 deliveries when estimated using the US-CDC’s SMM algorithm; 3.12% (n = 200,090) when estimated using the ACOG’s gold standard guidelines; 0.31% (n = 20,084) when estimated using Zwart et al.’s SMM criteria; and 1.36% (n = 87,452) when estimated using the EURONET-SAMM indicators.

**Table 1:** Severe maternal morbidity incidence using four indicators and the study populations’ characteristics (2003–2018)

	US-CDC’s SMM					Gold standard guideline for SMM^a^					Zwart et al.’s SMM					EURONET-SAMM				
	No (n = 6,269,558)		Yes (n = 151,533)		*P*-value	No (n = 6,221,001)		Yes (n = 200,090)		*P*-value	No (n = 6,401,007)		Yes (n = 20,084)		*P*-value	No (n = 6,333,639)		Yes (n = 87,452)		*P*-value
	N	%	N	%		N	%	N	%		N	%	N	%		N	%	N	%	
Maternal age (y)																				
15–19	23,179	96.4	862	3.6	< .0001	22,944	95.4	1,097	4.6	< .0001	23,983	99.8	58	0.2	< .0001	23,664	98.4	377	1.6	< .0001
20–24	313,014	97.8	6,999	2.2		310,163	96.9	9,850	3.1		319,343	99.8	670	0.2		316,158	98.8	3,855	1.2	
25–29	1,757,569	98.1	34,153	1.9		1,743,220	97.3	48,502	2.7		1,787,457	99.8	4,265	0.2		1,771,009	98.8	20,713	1.2	
30–34	2,921,132	97.8	65,872	2.2		2,899,720	97.1	87,284	2.9		2,978,744	99.7	8,260	0.3		2,948,384	98.7	38,620	1.3	
35–39	1,100,678	96.9	35,550	3.1		1,092,320	96.1	43,908	3.9		1,130,918	99.5	5,310	0.5		1,116,714	98.3	19,514	1.7	
40–44	148,938	95.1	7,703	4.9		147,636	94.3	9,005	5.8		155,204	99.1	1,437	0.9		152,476	97.3	4,165	2.7	
45 +	5,048	92.8	394	7.2		4,998	91.8	444	8.2		5,358	98.5	84	1.5		5,234	96.2	208	3.8	
Household income																				
1Q (Low)	1,257,640	97.5	32,705	2.5	< .0001	1,247,497	96.7	42,848	3.3	< .0001	1,285,885	99.7	4,460	0.4	< .0001	1,271,599	98.6	18,746	1.5	< .0001
2Q	1,590,797	97.7	38,155	2.3		1,578,391	96.9	50,561	3.1		1,623,749	99.7	5,203	0.3		1,606,787	98.6	22,165	1.4	
3Q	2,211,770	97.7	51,557	2.3		2,194,855	97	68,472	3		2,256,625	99.7	6,702	0.3		2,233,451	98.7	29,876	1.3	
4Q (High)	1,209,351	97.7	29,116	2.4		1,200,258	96.9	38,209	3.1		1,234,748	99.7	3,719	0.3		1,221,802	98.7	16,665	1.4	
Type of health insurance																				
Self-employed	1,689,352	97.3	46,924	2.7	< .0001	1,675,974	96.5	60,302	3.5	< .0001	1,729,256	99.6	7,020	0.4	< .0001	1,710,475	98.5	25,801	1.5	< .0001
Employee	4,546,866	97.8	102,663	2.2		4,512,048	97	137,481	3		4,636,691	99.7	12,838	0.3		4,588,734	98.7	60,795	1.3	
Medical aid	33,340	94.5	1,946	5.5		32,979	93.5	2,307	6.5		35,060	99.4	226	0.6		34,430	97.6	856	2.4	
Residential area																				
Seoul	1,281,035	97.7	29,985	2.3	< .0001	1,271,383	97	39,637	3	< .0001	1,307,052	99.7	3,968	0.3	< .0001	1,295,457	98.8	15,563	1.2	< .0001
Metropolitan region	1,544,941	97.6	37,502	2.4		1,533,021	96.9	49,422	3.1		1,576,887	99.7	5,556	0.4		1,557,354	98.4	25,089	1.6	
Small cities	3,027,116	97.7	72,423	2.3		3,003,602	96.9	95,937	3.1		3,090,716	99.7	8,823	0.3		3,059,769	98.7	39,770	1.3	
Rural	416,466	97.3	11,623	2.7		412,995	96.5	15,094	3.5		426,352	99.6	1,737	0.4		421,059	98.4	7,030	1.6	
Mode of delivery																				
Vaginal delivery	2,170,826	98.8	27,032	1.2	< .0001	2,155,660	98.1	42,198	1.9	< .0001	2,194,396	99.8	3,462	0.2	< .0001	2,177,073	99.1	20,785	1	< .0001
Instrument delivery	1,750,562	98	35,350	2		1,736,117	97.2	49,795	2.8		1,782,129	99.8	3,783	0.2		1,758,974	98.5	26,938	1.5	
Cesarean section	2,348,170	96.3	89,151	3.7		2,329,224	95.6	108,097	4.4		2,424,482	99.5	12,839	0.5		2,397,592	98.4	39,729	1.6	
Preterm birth																				
No	6,139,508	97.8	138,641	2.2	< .0001	6,092,686	97.1	185,463	3	< .0001	6,259,755	99.7	18,394	0.3	< .0001	6,196,081	98.7	82,068	1.3	< .0001
Yes	130,050	91	12,892	9		128,315	89.8	14,627	10.2		141,252	98.8	1,690	1.2		137,558	96.2	5,384	3.8	
Parity																				
0	3,248,026	97.4	86,637	2.6	< .0001	3,219,016	96.5	115,647	3.5	< .0001	3,323,886	99.7	10,777	0.3	< .0001	3,283,131	98.5	51,532	1.6	< .0001
1 +	3,021,532	97.9	64,896	2.1		3,001,985	97.3	84,443	2.7		3,077,121	99.7	9,307	0.3		3,050,508	98.8	35,920	1.2	
Multiple birth																				
No	6,186,857	97.8	141,715	2.2	< .0001	6,139,428	97	189,144	3	< .0001	6,309,615	99.7	18,957	0.3	< .0001	6,244,363	98.7	84,209	1.3	< .0001
Yes	82,701	89.4	9,818	10.6		81,573	88.2	10,946	11.8		91,392	98.8	1,127	1.2		89,276	96.5	3,243	3.5	
Prenatal care^*^																				
Adequate	5,309,789	97.8	122,423	2.3	< .0001	5,267,814	97	164,398	3	< .0001	5,416,867	99.7	15,345	0.3	< .0001	5,358,203	98.6	74,009	1.4	< .0001
Intermediate	871,738	97.1	25,838	2.9		865,763	96.5	31,813	3.5		893,375	99.5	4,201	0.5		885,419	98.7	12,157	1.4	
Inadequate	57,314	96.6	2,006	3.4		56,861	95.9	2,459	4.2		58,990	99.4	330	0.6		58,436	98.5	884	1.5	
Missing	31,983					31,983					31,983					31,983				
Maternal comorbidities^†^																				
0	3,908,136	98.2	73,223	1.8	< .0001	3,880,064	97.5	101,295	2.5	< .0001	3,972,890	99.8	8,469	0.2	< .0001	3,935,912	98.9	45,447	1.1	< .0001
1 +	2,361,422	96.8	78,310	3.2		2,340,937	96	98,795	4.1		2,428,117	99.5	11,615	0.5		2,397,727	98.3	42,005	1.7	
Type of hospital																				
≥ 500 beds	341,950	89.8	38,680	10.2	< .0001	337,394	88.6	43,236	11.4	< .0001	376,134	98.8	4,496	1.2	< .0001	366,186	96.2	14,444	3.8	< .0001
100–499 beds	669,389	95.2	33,786	4.8		661,773	94.1	41,402	5.9		697,438	99.2	5,737	0.8		690,670	98.2	12,505	1.8	
30–99 beds	2,513,725	98.5	37,579	1.5		2,496,628	97.9	54,676	2.1		2,545,664	99.8	5,640	0.2		2,518,029	98.7	33,275	1.3	
< 30 beds	2,744,494	98.5	41,488	1.5		2,725,206	97.8	60,776	2.2		2,781,771	99.9	4,211	0.2		2,758,754	99	27,228	1	
Region of hospital																				
Seoul	1,294,441	97.3	35,505	2.7	< .0001	1,284,473	96.6	45,473	3.4	< .0001	1,325,181	99.6	4,765	0.4	< .0001	1,312,693	98.7	17,253	1.3	< .0001
Metropolitans	1,747,196	97.5	45,328	2.5		1,733,515	96.7	59,009	3.3		1,785,397	99.6	7,127	0.4		1,761,969	98.3	30,555	1.7	
Small cities	3,174,813	97.9	69,582	2.1		3,150,354	97.1	94,041	2.9		3,236,279	99.8	8,116	0.3		3,205,299	98.8	39,096	1.2	
Rural	53,108	97.9	1,118	2.1		52,659	97.1	1,567	2.9		54,150	99.9	76	0.1		53,678	99	548	1	
Total	6,269,558	97.6	151,533	2.4		6,221,001	96.9	200,090	3.1		6,401,007	99.7	20,084	0.3		6,333,639	98.6	87,452	1.4	

*Prenatal care is estimated using Kessner’s adequate prenatal care index

^†^Maternal comorbidities included are: cardiac disease, renal disease, musculoskeletal disease, digestive disorder, blood disease, mental disorders, CNS disease, rheumatic heart disease, placentation disorder, chronic hypertension, pregnancy hypertension, lupus, collagen vascular disorder, rheumatoid arthritis, diabetes, diabetes complicating pregnancy, obesity, and asthma/chronic bronchitis

*EURONET-SAMM* European Network on Severe Acute Maternal Morbidity, *SAMM* severe acute maternal morbidity, *SMM* severe maternal morbidity, *US-CDC* United States Centers for Disease Control and Prevention

^a^Excluding hospital length of stay

Table 2 outlines the frequency and fractions of the sub-indicators constituting each of the SMM indicators. The highest incidence estimated using the US-CDC’s SMM algorithm was blood transfusion (77.3%); when using the ACOG’s gold standard guidelines, it was the SMM cases including blood transfusion (75.7%); when using Zwart et al.’s indicators it was ICU admission (50%) and obstetric hemorrhage (34%); and when using the EURONET-SAMM indicators it was obstetric hemorrhage (72.3%).

**Table 2:** The incidence of severe maternal morbidity using the four indicators

	US-CDC’s SMM		Gold standard guideline for SMM^a^		Zwart et al.’s SMM		EURONET-SAMM	
	(n = 151,533)		(n = 200,090)		(n = 20,084)		(n = 87,452)	
	N	%	N	%	N	%	N	%
Acute myocardial infarction	645	0.4						
Aneurysm	66	0						
Acute renal failure	2310	1.5						
Adult respiratory distress syndrome	610	0.4						
Amniotic fluid embolism	233	0.2						
Cardiac arrest/ ventricular fibrillation	293	0.2						
Conversion of cardiac rhythm	744	0.5						
Disseminated intravascular coagulation	15,131	10						
Eclampsia	4912	3.2						
Heart failure/arrest during procedure or surgery	5	0						
Puerperal cerebrovascular disorders	1330	0.9						
Pulmonary edema/ acute heart failure	5283	3.5						
Severe anesthesia complications	140	0.1						
Sepsis	12,016	7.9						
Shock	7798	5.2						
Sickle cell anemia with crisis	5	0						
Air and thrombotic embolism	1821	1.2						
Blood product transfusion	117,076	77.3						
Hysterectomy	7251	4.8						
Temporary tracheostomy	84	0.1						
Ventilation	3233	2.1						
US-CDC’s SMM			151,533	75.7				
Prolonged postpartum length of stay*			–	–				
Maternal intensive care unit admission			10,188	5.1				
Administration of blood products (≥ 4 units)			636	0.3				
30 days readmission			50,265	25.1				
ICU admission					10,188	50.7		
Uterine rupture					637	3.2		
Eclampsia/HELLP syndrome					5406	26.9		
Major obstetric hemorrhage (≥ 4 units of blood cells; embolization or hysterectomy for MOH)					6837	34.0		
Miscellaneous (opinion of the treating obstetrician) *					–	–		
Eclampsia							7302	8.4
Septicemia during pregnancy or labor							15,672	17.9
Hysterectomy in the context of pregnancy							7251	8.3
Hysterectomy associated with obstetric hemorrhage							5839	6.7
Red blood cell transfusion associated with OH							63,213	72.3

*Excluded in the indicators

*EURONET-SAMM* European Network on Severe Acute Maternal Morbidity, *GSG* gold standard guidelines (American College of Obstetricians and Gynecologists) for SMM, *HELLP* hemolysis, elevated liver enzymes and low platelet syndrome, *ICU* intensive care unit, *MOH* major obstetric hemorrhage, *OH* obstetric hemorrhage, *SMM* severe maternal morbidity, *US-CDC* United States Centers for Disease Control and Prevention

Table 3 shows the relationships between the risk factors and the risk of SMM. All four SMM indicators showed that advanced maternal age significantly increased the risk of SMM compared to the reference group (US-CDC, age 40–44 years: RR 1.67, 95% CI 1.63–1.71; age ≥ 45 years: RR 1.85, 95% CI 1.67–2.04; ACOG gold standard guideline, age 40–44 years: RR 1.52, 95% CI 1.49–1.56; age ≥ 45 years: RR 1.70, 95% CI 1.54–1.87; Zwart’s indicators, 40–44 years: RR 2.72, 95% CI 2.55–2.90, age ≥ 45 year: RR 3.19, 95% CI 2.56–3.97; EURONET indicators, 40–44 years: RR 2.04, 95% CI 1.97–2.11, age ≥ 45 year: RR 2.49; 95% CI 2.17–2.86). Moreover, there was a J-shaped curve relation between maternal age and risk of SMM in the US-CDC and ACOG SMM indicators. Women who received inadequate prenatal care had a higher risk of SMM than those who received adequate prenatal care (US-CDC: RR 1.39, 95% CI 1.33–1.45; ACOG gold standard guideline: RR 1.26, 95% CI 1.2–1.31; Zwart et al.: RR 1.38, 95% CI 1.23–1.54; EURONET: RR 1.10, 95% CI 1.03–1.17). In addition, women who had intermediate prenatal care had 5–25% higher risk of SMM compared with those who received adequate prenatal care with all indicators except EURONET SMM (US-CDC: RR 1.13, 95% CI 1.12–1.15; ACOG gold standard guideline: RR 1.05, 95% CI 1.04–1.06; Zwart et al.: RR 1.10, 95% CI 1.06–1.14). Women who lived in rural areas had a higher risk of SMM than those who lived in Seoul (US-CDC: RR 1.21, 95% CI 1.18–1.24; ACOG gold standard guideline: RR 1.17 95% CI 1.14–1.20, Zwart et al.: RR 1.49, 95% CI 1.39–1.60; and EURONET: RR 1.39, 95% CI 1.34–1.44).

**Table 3:** The relationship between risk factors and the four indicators of severe maternal morbidity

	US-CDC’s SMM			P-value	Gold standard guideline for SMM			P-value	Zwart’s SMM			P-value	EURONET-SAMM			P-value
	RR	95% CI			RR	95% CI			RR	95% CI			RR	95% CI		
Individual factors																
Maternal age (y)																
15–19	1.35	1.25	1.45	< .0001	1.27	1.19	1.36	< .0001	0.85	0.64	1.13	0.277	1.05	0.95	1.17	0.3392
20–24	1.09	1.06	1.12	< .0001	1.09	1.06	1.11	< .0001	0.88	0.81	0.95	0.0024	0.98	0.95	1.02	0.2914
25–29	1.00				1.00				1.00				1.00			
30–34	1.10	1.08	1.11	< .0001	1.05	1.04	1.07	< .0001	1.17	1.12	1.21	< .0001	1.14	1.12	1.16	< .0001
35–39	1.34	1.32	1.36	< .0001	1.24	1.23	1.26	< .0001	1.76	1.69	1.84	< .0001	1.47	1.44	1.50	< .0001
40–44	1.67	1.63	1.71	< .0001	1.52	1.49	1.56	< .0001	2.72	2.55	2.90	< .0001	2.04	1.97	2.11	< .0001
≥ 45	1.85	1.67	2.04	< .0001	1.70	1.54	1.87	< .0001	3.19	2.56	3.97	< .0001	2.49	2.17	2.86	< .0001
Household Income level																
1Q (Low)	1.17	1.15	1.19	< .0001	1.14	1.12	1.16	< .0001	1.30	1.24	1.36	< .0001	1.13	1.10	1.15	< .0001
2Q	1.14	1.13	1.16	< .0001	1.12	1.10	1.13	< .0001	1.24	1.18	1.29	< .0001	1.09	1.07	1.12	< .0001
3Q	1.07	1.06	1.09	< .0001	1.06	1.05	1.08	< .0001	1.11	1.07	1.16	< .0001	1.04	1.02	1.06	< .0001
4Q (High)	1.00				1.00				1.00				1.00			
Type of health insurance																
Self-employed	1.14	1.13	1.16	< .0001	1.12	1.11	1.13	< .0001	1.21	1.17	1.25	< .0001	1.14	1.12	1.15	< .0001
Employees	1.00				1.00				1.00				1.00			
Medical aid	1.80	1.71	1.89	< .0001	1.70	1.63	1.78	< .0001	1.58	1.37	1.81	< .0001	1.57	1.46	1.68	< .0001
Residential areas																
Seoul	1.00				1.00				1.00				1.00			
Metropolitans	1.05	1.03	1.08	< .0001	1.04	1.01	1.06	0.001	1.00	0.93	1.06	0.9168	1.05	1.02	1.08	0.0038
Small cities	1.15	1.13	1.18	< .0001	1.12	1.10	1.14	< .0001	1.22	1.15	1.28	< .0001	1.15	1.12	1.18	< .0001
Rural	1.21	1.18	1.24	< .0001	1.17	1.14	1.20	< .0001	1.49	1.39	1.60	< .0001	1.39	1.34	1.44	< .0001
Obstetric factors																
Mode of delivery																
Vaginal delivery	1.00				1.00				1.00				1.00			
Instrument delivery	1.53	1.51	1.56	< .0001	1.38	1.36	1.40	< .0001	1.37	1.30	1.43	< .0001	1.49	1.46	1.52	< .0001
Cesarean section	2.28	2.25	2.32	< .0001	1.84	1.82	1.86	< .0001	2.51	2.41	2.61	< .0001	1.41	1.39	1.44	< .0001
Preterm birth																
No	1.00				1.00				1.00				1.00			
Yes	1.39	1.36	1.42	< .0001	1.36	1.34	1.39	< .0001	1.61	1.53	1.70	< .0001	1.53	1.49	1.58	< .0001
Parity																
0	1.25	1.24	1.27	< .0001	1.27	1.26	1.28	< .0001	1.15	1.12	1.19	< .0001	1.39	1.37	1.41	< .0001
1 +	1.00				1.00				1.00				1.00			
Multiple birth																
No	1.00				1.00				1.00				1.00			
Yes	1.64	1.60	1.67	< .0001	1.59	1.56	1.62	< .0001	1.37	1.29	1.46	< .0001	1.37	1.32	1.42	< .0001
Prenatal care^*^																
Adequate	1.00				1.00				1.00				1.00			
Intermediate	1.13	1.12	1.15	< .0001	1.05	1.04	1.06	< .0001	1.10	1.06	1.14	< .0001	0.97	0.95	0.99	0.0032
Inadequate	1.39	1.33	1.45	< .0001	1.26	1.21	1.31	< .0001	1.38	1.23	1.54	< .0001	1.10	1.03	1.17	0.0066
Maternal comorbidities^†^																
0	1.00				1.00				1.00				1.00			
≥ 1	1.51	1.50	1.53	< .0001	1.42	1.41	1.43	< .0001	2.32	2.25	2.39	< .0001	1.33	1.31	1.35	< .0001
Provision factors																
Type of hospital																
≥ 500 beds	5.54	5.45	5.62	< .0001	4.47	4.40	4.53	< .0001	3.29	3.15	3.44	< .0001	2.53	2.48	2.59	< .0001
100–499 beds	2.94	2.89	2.99	< .0001	2.54	2.50	2.57	< .0001	2.61	2.50	2.71	< .0001	1.35	1.32	1.38	< .0001
30–99 beds	1.00				1.00				1.00				1.00			
< 30 beds	1.04	1.03	1.06	< .0001	1.04	1.03	1.06	< .0001	0.69	0.66	0.72	< .0001	0.81	0.80	0.83	< .0001
Region of hospital																
Seoul	1.00				1.00				1.00				1.00			
Metropolitan	1.35	1.32	1.38	< .0001	1.32	1.30	1.35	< .0001	1.60	1.51	1.69	< .0001	1.52	1.47	1.57	< .0001
Small cities	1.23	1.21	1.25	< .0001	1.22	1.20	1.24	< .0001	1.02	0.97	1.07	0.4545	1.12	1.09	1.15	< .0001
Rural	1.17	1.10	1.24	< 0.0001	1.19	1.13	1.26	< .0001	0.47	0.38	0.60	< .0001	0.92	0.84	1.01	0.0704

Adjusted for maternal age, household income level, type of health insurance, residential area, mode of delivery, preterm birth, parity, multiple birth, prenatal care, maternal comorbidities, type of hospital, region of hospital, and year

*EURONET-SAMM* European Network on Severe Acute Maternal Morbidity, *US-CDC* United States Centers for Disease Control and Prevention, *CI* confidence interval, *GSG* gold standard guidelines, *RR* relative risk, *SMM* severe maternal morbidity

*Prenatal care is estimated using Kessner’s adequate prenatal care index

^†^Maternal comorbidities included are: cardiac disease, renal disease, musculoskeletal disease, digestive disorder, blood disease, mental disorders, CNS disease, rheumatic heart disease, placentation disorder, chronic hypertension, pregnancy hypertension, lupus, collagen vascular disorder, rheumatoid arthritis, diabetes, diabetes complicating pregnancy, obesity, and asthma/chronic bronchitis

## Discussion

This study compared the SMM risk assessment in a South Korean population estimated by using each of the four international indicators and identified the factors underlying the differences among the risk assessments obtained by the various indicators. The SMM rate during 16 years in South Korea differed depending on the sub-indicator composition of each indicator. There are several factors that explain why different SMM risk estimates can be obtained, though in the very same population, by using different indicators. First, differences in the SMM rate may be due to varying sub-indicator severity levels. In the US, for example, the scope of SMM is broader, since it includes past near-miss events. Conversely, the EURONET-SAMM indicators adopted by European countries mainly include acute-phase or high-severity SMM events [9, 10]. Second, this study verified the differences in SMM rate between South Korea and other countries using the same SMM indicators. When the US-CDC SMM algorithm was applied, a similar incidence rate to that of Howell et al. (2.5% and 2.4%) was found [17, 18]. However, the application of the ACOG gold standard guidelines [19] resulted in an incidence rate of 2%, and when using Zwart et al.’s indicators, [9] the incidence rate was 1.7%, indicating significant differences. These differences may be due to the length of the postnatal hospital stay sub-indicator from the ACOG indicators, which was excluded from this study when adapting the indicator to the South Korean situation, given that > 50% of deliveries in this study’s population would have been classified as SMM events had the US cut-off point been applied (Additional file 1). Moreover, one of Zwart et al.’s sub-indicators was that any postnatal condition deemed to be severe by an obstetrics and gynecology specialist was classified as SMM, in addition to their other listed sub-indicators. This sub-indicator was also excluded from the analysis in this study because there were no clear-cut criteria for decision-making. This may also have contributed to differences in the SMM rate estimations. Third, ethnocultural differences between Caucasians and Koreans and the representativeness of data may also have contributed to differences in the SMM incidence rates. Moreover, when tracking the deliveries of South Korean women over a 16-year period using the US-CDC SMM algorithm, some indicators were associated with diagnostic codes in < 100 cases or those with four procedure codes, especially those associated with < 10 cases in total. For example, sickle cell anemia, which occurred in five of a total of 6.4 million deliveries over a 16-year period, is known to be a disease that frequently occurs in people of African ethnicity. This example highlights the need for further in-depth studies concerning the adequacy of such indicators with little relevance to the domestic population as an indicator of maternal health risk in a largely single-ethnic Asian country, such as South Korea.

Furthermore, the greater the difference between the frequency of the SMM incidence according to each indicator and that of the sum of its sub-indicators, the higher the likelihood of overlapping sub-indicators, which indicates the presence of prenatal complication cases. Considering the high frequency of SMM due to obstetric hemorrhage or the blood transfusions needed for treatment, further research is needed to investigate the conditions that lead to blood transfusions.

The differences in maternal age and patterns of SMM risk among individual SMM indicators were also analyzed. Using US-CDC and ACOG SMM indicators, the risk of SMM increased as maternal age decreased or increased in relation to the reference age group (25–29 years), following a J-shaped curve. In particular, the risk of SMM was significantly higher in the > 35-year age group than in the < 35-year age group. In a previous study in which 403,116 deliveries in New York State hospitals were analyzed using the US-CDC SMM indicators, women in their teens, 30 s, and 40 s had a 1.28-, 1.09-, and 1.48-fold risk of SMM, respectively, compared with SMM risk in those in their 20 s, forming an age-dependent J-shaped curve. A high risk of SMM has been reported, showing a J-shaped curve according to age [20]. In the Zwart et al.’s and EURONET indicators, there was no statistically significant association between the 15–24-year age group and the risk of SMM. Furthermore, the 35–39-year age group had approximately a 1.8-fold risk of SMM, that is, only a slightly increased risk compared to that of the 30–34-year age group. The 40–45-year age group had a twofold risk of SMM compared with the reference group. Using Zwart et al.’s indicators, the ≥ 45-year age group, in particular, had a > threefold risk of SMM compared with the reference group. This result might be influenced by the composition of the four different SMM indicators. For example, Zwart et al.’s indicator consists of more acute and severe descriptors of SMM, such as ICU admission or cases of transfusion of ≥ 4 units of blood, [9] compared to other SMM indicators, thus yielding a higher risk of SMM with an advanced maternal age, which is a risk factor for maternal health when compared with a younger maternal age. There may be differences in the significance of the age effect (for teens, in particular) on SMM depending on the indicator; however, in the case of an advanced-age pregnancy, the risk increased in all four SMM indicators, as shown in this study.

Women who had inadequate prenatal care had a significant 1.1–1.4-times higher risk of SMM compared with those who had adequate prenatal care. Similarly, pregnant mothers who received an insufficient level of prenatal care, if not inadequate care, also had a higher risk of SMM (range 5–25%) compared with those who received adequate prenatal care. Although not analyzed in this study, considering that underlying disease is a very high-risk factor for SMM and can be a determinant of the delivery mode, appropriate prenatal management is likely to be a contributing factor for prevention of SMM. In this context, there is a need for continuous support for strategic programs to ensure adequate prenatal management.

Interestingly, considering socioeconomic status, the SMM risk of mothers living in rural areas was 1.2- to 1.5-fold higher than that of mothers living in Seoul. This finding, that socio-economic factors were closely correlated with the risk of SMM, is consistent with that of a previous study [20, 21] in which the reason for a higher risk of SMM in women of African-American ethnicity compared with those of European ethnicity was due to differences in health-care service quality between hospitals located in their respective regions, whereby the higher the patient fraction of non-Europeans and the higher the fraction of medical beneficiaries, the higher the risk of SMM [20]. As another underlying mechanism for this difference; it has been reported that rural areas have more limited access to health care. The number of doctors practicing in rural areas is lower than that in urban areas, [20] and rural residents have less chance of accessing the nearest hospital with a maternity unit within a 30-min driving distance [21]. The disparity of health-care resources between regions in South Korea may be a contributing factor to the higher risk of SMM in pregnant women living in rural areas.

This study had some limitations. First, we used claims data; therefore, it was not possible to identify those outside NHI coverage, which might have led to an underestimation of the outcomes. Despite this limitation, our results can be considered reliable because we analyzed the entire target population through a population-based large-scale cohort study with long-term follow-up of all pregnant mothers in South Korea. Furthermore, some sub-indicators were excluded when selecting the codes of each indicator, which might have led to corresponding underestimations. For example, the ACOG indicator regarding the postnatal hospital stay was excluded from the analysis of this study because estimations according to the US indicators would have resulted in classification of > 50% of deliveries of South Korean women as SMM events. Furthermore, Zwart et al.’s indicator that other SMMs could be classified as SMM, according to the opinion of the consulting obstetrician, could not be considered in this study due to a lack of reference for the related decision-making. Second, due to the limited availability of data, we could not correct for important risk factors affecting the development of SMM (e.g., education level, gestational age, and number of weeks for preterm births). Moreover, the accuracy of the prenatal care adequacy check may have been impaired due to the calculation method. However, it was the only method that could be used to ascertain the prevention effect of adequate prenatal care based on the available data, and the results obtained can be considered meaningful despite this limitation, because it confirms the importance of prenatal management.

The strengths of this study are as follows. First, the results are representative, because an entire population was analyzed, i.e., South Korean women of reproductive age for a follow-up period spanning 16 years using delivery data from a large-scale childbirth cohort. Second, this study is the first to have compared the risk of SMM using various international SMM indicators. Moreover, the study findings provide epidemiological, clinical, and policy-related basic data to investigate maternal health indicators tailored to the South Korean situation. Third, not only was the SMM incidence identified, but it was also shown to be affected by socio-demographic factors, such as age, income, and residential area; obstetric factors, such as preterm birth and multiple births; and provider factors. Thus, the results of this study provide a basis for policy development aimed to prevent SMM in the future. Particularly, adequate prenatal care as a preventable factor highlights the need to further investigate preventable or predictable factors in the future. Finally, while it may be useful to assess the quality of maternal health using high-quality indicators developed in other countries, this study shows the importance of developing maternal health quality indicators sensitive to country-specific ethnocultural characteristics. In this respect, the results of this study are likely to serve as a useful basis for further research aimed at promoting maternal health.

## Conclusions

This study found that SMM was associated with maternal age, socioeconomic status and obstetric factors using four international SMM indicators. In particular, blood transfusions were significantly involved in the SMM events; therefore, more studies are needed to identify the conditions leading to blood transfusions, as well as other SMM-prevention factors.

## Supplementary Information

**Additional file 1.** The result of Chi Square test on severe maternal morbidity incidence using four indicators and the study populations’ characteristics (2003 to 2018).

## Data Availability

Data was obtained from the National Health Insurance Sharing Service and are available from https://nhiss.nhis.or.kr/bd/ab/bdaba000eng.do with the permission of the National Health Insurance Sharing Service.

## Abbreviations

ACOG: American College of Obstetricians and Gynecologists
CI: Confidence interval
EURONET-SAMM: European Network on Severe Acute Maternal Morbidity
GSG: Gold standard guidelines (American College of Obstetricians and Gynecologists)
HELLP: Hemolysis, elevated liver enzymes and low platelet syndrome
ICU: Intensive care unit
MOH: Major obstetric hemorrhage
MMR: Maternal mortality ratio
NHI: National Health Insurance
OH: Obstetric hemorrhage
RR: Relative risk
SAMM: Severe acute maternal morbidity
SMM: Severe maternal morbidity
US-CDC: United States Centers for Disease Control and Prevention
WHO: World Health Organization
