# Supplementary material for: Comparison of global indicators for severe maternal morbidity among South Korean women who delivered from 2003 to 2018: a population-based retrospective cohort study
Source: Reprod Health. 2022 Aug 13;19:177. doi: 10.1186/s12978-022-01482-y (PMC9375335; doi:10.1186/s12978-022-01482-y)
Supplement: Supplementary file 1 — Additional file 1. The result of Chi Square test on severe maternal morbidity incidence using four indicators and the study populations’ characteristics (2003 to 2018). [file 12978_2022_1482_MOESM1_ESM.docx]

Additional file 1. The result of Chi Square test on severe maternal morbidity incidence using four indicators and the study populations’ characteristics (2003 to 2018)

|  |  | **US-CDC's SMM** | | | | | |  | **Gold standard guideline for SMM^a^** | | | | | | |  | **Zwart et al.'s SMM** | | | | | | |  | **EURONET-SAMM** | | | | | | | |  |
| --- | --- | --- | --- | --- | --- | --- | --- | --- | --- | --- | --- | --- | --- | --- | --- | --- | --- | --- | --- | --- | --- | --- | --- | --- | --- | --- | --- | --- | --- | --- | --- | --- | --- |
|  | No  (n = 6,269,558) | | | Yes  (n = 151,533) | | *χ ^2^ (d)* | *P*-value | No  (n = 6,221,001) | | | Yes  (n = 200,090) | | *χ ^2^ (d)* | *P-*value | No  (n = 6,401,007) | | | | Yes  (n = 20,084) | | *χ ^2^ (d)* | *P-*value | | No  (n = 6,333,639) | | | | Yes  (n = 87,452) | | | *χ ^2^ (d)* | *P-*value |  |
|  | N | | % | N | % |  |  | N | | % | N | % |  |  | N | | | % | N | % |  |  | N | | | % | N | | % |  | |  |  |
| **Maternal age (y)** | | |  |  |  |  |  |  | |  |  |  |  |  |  | | |  |  |  |  |  |  | | |  |  | |  |  | |  |  |
| 15-19 | 23,179 | | 96.4 | 862 | 3.6 | 10033.68(6) | <.0001 | 22,944 | | 95.4 | 1,097 | 4.6 | 7695.55(6) | <.0001 | 23,983 | | | 99.8 | 58 | 0.2 | 3531.83(6) | <.0001 | 23,664 | | | 98.4 | 377 | | 1.6 | 4013.42(6) | | <.0001 |  |
| 20-24 | 313,014 | | 97.8 | 6,999 | 2.2 |  |  | 310,163 | | 96.9 | 9,850 | 3.1 |  |  | 319,343 | | | 99.8 | 670 | 0.2 |  |  | 316,158 | | | 98.8 | 3,855 | | 1.2 |  | |  |  |
| 25-29 | 1,757,569 | | 98.1 | 34,153 | 1.9 |  |  | 1,743,220 | | 97.3 | 48,502 | 2.7 |  |  | 1,787,457 | | | 99.8 | 4,265 | 0.2 |  |  | 1,771,009 | | | 98.8 | 20,713 | | 1.2 |  | |  |  |
| 30-34 | 2,921,132 | | 97.8 | 65,872 | 2.2 |  |  | 2,899,720 | | 97.1 | 87,284 | 2.9 |  |  | 2,978,744 | | | 99.7 | 8,260 | 0.3 |  |  | 2,948,384 | | | 98.7 | 38,620 | | 1.3 |  | |  |  |
| 35-39 | 1,100,678 | | 96.9 | 35,550 | 3.1 |  |  | 1,092,320 | | 96.1 | 43,908 | 3.9 |  |  | 1,130,918 | | | 99.5 | 5,310 | 0.5 |  |  | 1,116,714 | | | 98.3 | 19,514 | | 1.7 |  | |  |  |
| 40-44 | 148,938 | | 95.1 | 7,703 | 4.9 |  |  | 147,636 | | 94.3 | 9,005 | 5.8 |  |  | 155,204 | | | 99.1 | 1,437 | 0.9 |  |  | 152,476 | | | 97.3 | 4,165 | | 2.7 |  | |  |  |
| 45+ | 5,048 | | 92.8 | 394 | 7.2 |  |  | 4,998 | | 91.8 | 444 | 8.2 |  |  | 5,358 | | | 98.5 | 84 | 1.5 |  |  | 5,234 | | | 96.2 | 208 | | 3.8 |  | |  |  |
| **Household income** | | |  |  |  |  |  |  | |  |  |  |  |  |  | | |  |  |  |  |  |  | | |  |  | |  |  | |  |  |
| 1Q (Low) | 1,257,640 | | 97.5 | 32,705 | 2.5 | 239.51(3) | <.0001 | 1,247,497 | | 96.7 | 42,848 | 3.3 | 245.41(3) | <.0001 | 1,285,885 | | | 99.7 | 4,460 | 0.4 | 73.35(3) | <.0001 | 1,271,599 | | | 98.6 | 18,746 | | 1.5 | 111.38(3) | | <.0001 |  |
| 2Q | 1,590,797 | | 97.7 | 38,155 | 2.3 |  |  | 1,578,391 | | 96.9 | 50,561 | 3.1 |  |  | 1,623,749 | | | 99.7 | 5,203 | 0.3 |  |  | 1,606,787 | | | 98.6 | 22,165 | | 1.4 |  | |  |  |
| 3Q | 2,211,770 | | 97.7 | 51,557 | 2.3 |  |  | 2,194,855 | | 97 | 68,472 | 3 |  |  | 2,256,625 | | | 99.7 | 6,702 | 0.3 |  |  | 2,233,451 | | | 98.7 | 29,876 | | 1.3 |  | |  |  |
| 4Q (High) | 1,209,351 | | 97.7 | 29,116 | 2.4 |  |  | 1,200,258 | | 96.9 | 38,209 | 3.1 |  |  | 1,234,748 | | | 99.7 | 3,719 | 0.3 |  |  | 1,221,802 | | | 98.7 | 16,665 | | 1.4 |  | |  |  |
| **Type of health insurance** | | | |  |  |  |  |  | |  |  |  |  |  |  | | |  |  |  |  |  |  | | |  |  | |  |  | |  |  |
| Self-employed | 1,689,352 | | 97.3 | 46,924 | 2.7 | 2874.52(2) | <.0001 | 1,675,974 | | 96.5 | 60,302 | 3.5 | 2491.83(2) | <.0001 | 1,729,256 | | | 99.6 | 7,020 | 0.4 | 788.55(2) | <.0001 | 1,710,475 | | | 98.5 | 25,801 | | 1.5 | 598.62(2) | | <.0001 |  |
| Employee | 4,546,866 | | 97.8 | 102,663 | 2.2 |  |  | 4,512,048 | | 97 | 137,481 | 3 |  |  | 4,636,691 | | | 99.7 | 12,838 | 0.3 |  |  | 4,588,734 | | | 98.7 | 60,795 | | 1.3 |  | |  |  |
| Medical aid | 33,340 | | 94.5 | 1,946 | 5.5 |  |  | 32,979 | | 93.5 | 2,307 | 6.5 |  |  | 35,060 | | | 99.4 | 226 | 0.6 |  |  | 34,430 | | | 97.6 | 856 | | 2.4 |  | |  |  |
| **Residential area** | | |  |  |  |  |  |  | |  |  |  |  |  |  | | |  |  |  |  |  |  | | |  |  | |  |  | |  |  |
| Seoul | 1,281,035 | | 97.7 | 29,985 | 2.3 | 272.50(3) | <.0001 | 1,271,383 | | 97 | 39,637 | 3 | 280.21(3) | <.0001 | 1,307,052 | | | 99.7 | 3,968 | 0.3 | 276.15(3) | <.0001 | 1,295,457 | | | 98.8 | 15,563 | | 1.2 | 1280.56(3) | | <.0001 |  |
| Metropolitan region | 1,544,941 | | 97.6 | 37,502 | 2.4 |  |  | 1,533,021 | | 96.9 | 49,422 | 3.1 |  |  | 1,576,887 | | | 99.7 | 5,556 | 0.4 |  |  | 1,557,354 | | | 98.4 | 25,089 | | 1.6 |  | |  |  |
| Small cities | 3,027,116 | | 97.7 | 72,423 | 2.3 |  |  | 3,003,602 | | 96.9 | 95,937 | 3.1 |  |  | 3,090,716 | | | 99.7 | 8,823 | 0.3 |  |  | 3,059,769 | | | 98.7 | 39,770 | | 1.3 |  | |  |  |
| Rural | 416,466 | | 97.3 | 11,623 | 2.7 |  |  | 412,995 | | 96.5 | 15,094 | 3.5 |  |  | 426,352 | | | 99.6 | 1,737 | 0.4 |  |  | 421,059 | | | 98.4 | 7,030 | | 1.6 |  | |  |  |
| **Mode of delivery** | | |  |  |  |  |  |  | |  |  |  |  |  |  | | |  |  |  |  |  |  | | |  |  | |  |  | |  |  |
| Vaginal delivery | 2,170,826 | | 98.8 | 27,032 | 1.2 | 31118.13(2) | <.0001 | 2,155,660 | | 98.1 | 42,198 | 1.9 | 25096.71(2) | <.0001 | 2,194,396 | | | 99.8 | 3,462 | 0.2 | 5862.37(2) | <.0001 | 2,177,073 | | | 99.1 | 20,785 | | 1 | 4423.58(2) | | <.0001 |  |
| Instrument delivery | 1,750,562 | | 98 | 35,350 | 2 |  |  | 1,736,117 | | 97.2 | 49,795 | 2.8 |  |  | 1,782,129 | | | 99.8 | 3,783 | 0.2 |  |  | 1,758,974 | | | 98.5 | 26,938 | | 1.5 |  | |  |  |
| Cesarean section | 2,348,170 | | 96.3 | 89,151 | 3.7 |  |  | 2,329,224 | | 95.6 | 108,097 | 4.4 |  |  | 2,424,482 | | | 99.5 | 12,839 | 0.5 |  |  | 2,397,592 | | | 98.4 | 39,729 | | 1.6 |  | |  |  |
| **Preterm birth** | | |  |  |  |  |  |  | |  |  |  |  |  |  | | |  |  |  |  |  |  | | |  |  | |  |  | |  |  |
| No | 6,139,508 | | 97.8 | 138,641 | 2.2 | 28134.80(1) | <.0001 | 6,092,686 | | 97.1 | 185,463 | 3 | 24525.87(1) | <.0001 | 6,259,755 | | | 99.7 | 18,394 | 0.3 | 3544.96(1) | <.0001 | 6,196,081 | | | 98.7 | 82,068 | | 1.3 | 6292.49(1) | | <.0001 |  |
| Yes | 130,050 | | 91 | 12,892 | 9 |  |  | 128,315 | | 89.8 | 14,627 | 10.2 |  |  | 141,252 | | | 98.8 | 1,690 | 1.2 |  |  | 137,558 | | | 96.2 | 5,384 | | 3.8 |  | |  |  |
| **Parity** |  | |  |  |  |  |  |  | |  |  |  |  |  |  | | |  |  |  |  |  |  | | |  |  | |  |  | |  |  |
| 0 | 3,248,026 | | 97.4 | 86,637 | 2.6 | 1707.54(1) | <.0001 | 3,219,016 | | 96.5 | 115,647 | 3.5 | 2845.44(1) | <.0001 | 3,323,886 | | | 99.7 | 10,777 | 0.3 | 24.06(1) | <.0001 | 3,283,131 | | | 98.5 | 51,532 | | 1.6 | 1736.88(1) | | <.0001 |  |
| 1+ | 3,021,532 | | 97.9 | 64,896 | 2.1 |  |  | 3,001,985 | | 97.3 | 84,443 | 2.7 |  |  | 3,077,121 | | | 99.7 | 9,307 | 0.3 |  |  | 3,050,508 | | | 98.8 | 35,920 | | 1.2 |  | |  |  |
| **Multiple birth** | | |  |  |  |  |  |  | |  |  |  |  |  |  | | |  |  |  |  |  |  | | |  |  | |  |  | |  |  |
| No | 6,186,857 | | 97.8 | 141,715 | 2.2 | 27740.90(1) | <.0001 | 6,139,428 | | 97 | 189,144 | 3 | 23615.41(1) | <.0001 | 6,309,615 | | | 99.7 | 18,957 | 0.3 | 2467.65(1) | <.0001 | 6,244,363 | | | 98.7 | 84,209 | | 1.3 | 3209.85(1) | | <.0001 |  |
| Yes | 82,701 | | 89.4 | 9,818 | 10.6 |  |  | 81,573 | | 88.2 | 10,946 | 11.8 |  |  | 91,392 | | | 98.8 | 1,127 | 1.2 |  |  | 89,276 | | | 96.5 | 3,243 | | 3.5 |  | |  |  |
| **Prenatal care**^*^ | | |  |  |  |  |  |  | |  |  |  |  |  |  | | |  |  |  |  |  |  | | |  |  | |  |  | |  |  |
| Adequate | 5,309,789 | | 97.8 | 122,423 | 2.3 | 1586.60(2) | <.0001 | 5,267,814 | | 97 | 164,398 | 3 | 899.17(2) | <.0001 | 5,416,867 | | | 99.7 | 15,345 | 0.3 | 971.31(2) | <.0001 | 5,358,203 | | | 98.6 | 74,009 | | 1.4 | 7.64(2) | | <.0001 |  |
| Intermediate | 871,738 | | 97.1 | 25,838 | 2.9 |  |  | 865,763 | | 96.5 | 31,813 | 3.5 |  |  | 893,375 | | | 99.5 | 4,201 | 0.5 |  |  | 885,419 | | | 98.7 | 12,157 | | 1.4 |  | |  |  |
| Inadequate | 57,314 | | 96.6 | 2,006 | 3.4 |  |  | 56,861 | | 95.9 | 2,459 | 4.2 |  |  | 58,990 | | | 99.4 | 330 | 0.6 |  |  | 58,436 | | | 98.5 | 884 | | 1.5 |  | |  |  |
| Missing | 31,983 | |  |  |  |  |  | 31,983 | |  |  |  |  |  | 31,983 | | |  |  |  |  |  | 31,983 | | |  |  | |  |  | |  |  |
| **Maternal comorbidities**^†^ | | | |  |  |  |  |  | |  |  |  |  |  |  | | |  |  |  |  |  |  | | |  |  | |  |  | |  |  |
| 0 | 3,908,136 | | 98.2 | 73,223 | 1.8 | 12333.33(1) | <.0001 | 3,880,064 | | 97.5 | 101,295 | 2.5 | 11352.16(1) | <.0001 | 3,972,890 | | | 99.8 | 8,469 | 0.2 | 3365 (1) | <.0001 | 3,935,912 | | | 98.9 | 45,447 | | 1.1 | 3790.80(1) | | <.0001 |  |
| 1+ | 2,361,422 | | 96.8 | 78,310 | 3.2 |  |  | 2,340,937 | | 96 | 98,795 | 4.1 |  |  | 2,428,117 | | | 99.5 | 11,615 | 0.5 |  |  | 2,397,727 | | | 98.3 | 42,005 | | 1.7 |  | |  |  |
| **Type of hospital** | | |  |  |  |  |  |  | |  |  |  |  |  |  | | |  |  |  |  |  |  | | |  |  | |  |  | |  |  |
| ≥500 beds | 341,950 | | 89.8 | 38,680 | 10.2 | 136675(3) | <.0001 | 337,394 | | 88.6 | 43,236 | 11.4 | 119621(3) | <.0001 | 376,134 | | | 98.8 | 4,496 | 1.2 | 17936.63(3) | <.0001 | 366,186 | | | 96.2 | 14,444 | | 3.8 | 20808.17(3) | | <.0001 |  |
| 100-499 beds | 669,389 | | 95.2 | 33,786 | 4.8 |  |  | 661,773 | | 94.1 | 41,402 | 5.9 |  |  | 697,438 | | | 99.2 | 5,737 | 0.8 |  |  | 690,670 | | | 98.2 | 12,505 | | 1.8 |  | |  |  |
| 30-99 beds | 2,513,725 | | 98.5 | 37,579 | 1.5 |  |  | 2,496,628 | | 97.9 | 54,676 | 2.1 |  |  | 2,545,664 | | | 99.8 | 5,640 | 0.2 |  |  | 2,518,029 | | | 98.7 | 33,275 | | 1.3 |  | |  |  |
| <30 beds | 2,744,494 | | 98.5 | 41,488 | 1.5 |  |  | 2,725,206 | | 97.8 | 60,776 | 2.2 |  |  | 2,781,771 | | | 99.9 | 4,211 | 0.2 |  |  | 2,758,754 | | | 99 | 27,228 | | 1 |  | |  |  |
| **Region of hospital** | | |  |  |  |  |  |  | |  |  |  |  |  |  | | |  |  |  |  |  |  | | |  |  | |  |  | |  |  |
| Seoul | 1,294,441 | | 97.3 | 35,505 | 2.7 | 1448.61(3) | <.0001 | 1,284,473 | | 96.6 | 45,473 | 3.4 | 1105.93(3) | <.0001 | 1,325,181 | | | 99.6 | 4,765 | 0.4 | 961.80(3) | <.0001 | 1,312,693 | | | 98.7 | 17,253 | | 1.3 | 2252.34(3) | | <.0001 |  |
| Metropolitans | 1,747,196 | | 97.5 | 45,328 | 2.5 |  |  | 1,733,515 | | 96.7 | 59,009 | 3.3 |  |  | 1,785,397 | | | 99.6 | 7,127 | 0.4 |  |  | 1,761,969 | | | 98.3 | 30,555 | | 1.7 |  | |  |  |
| Small cities | 3,174,813 | | 97.9 | 69,582 | 2.1 |  |  | 3,150,354 | | 97.1 | 94,041 | 2.9 |  |  | 3,236,279 | | | 99.8 | 8,116 | 0.3 |  |  | 3,205,299 | | | 98.8 | 39,096 | | 1.2 |  | |  |  |
| Rural | 53,108 | | 97.9 | 1,118 | 2.1 |  |  | 52,659 | | 97.1 | 1,567 | 2.9 |  |  | 54,150 | | | 99.9 | 76 | 0.1 |  |  | 53,678 | | | 99 | 548 | | 1 |  | |  |  |
| Total | 6,269,558 | | 97.6 | 151,533 | 2.4 |  |  | 6,221,001 | | 96.9 | 200,090 | 3.1 |  |  | 6,401,007 | | | 99.7 | 20,084 | 0.3 |  |  | 6,333,639 | | | 98.6 | 87,452 | | 1.4 |  | |  |  |

^*^ Prenatal care is estimated using Kessner’s adequate prenatal care index.
^†^ Maternal comorbidities included are: cardiac disease, renal disease, musculoskeletal disease, digestive disorder, blood disease, mental disorders, CNS disease, rheumatic heart disease, placentation disorder, chronic hypertension, pregnancy hypertension, lupus, collagen vascular disorder, rheumatoid arthritis, diabetes, diabetes complicating pregnancy, obesity, and asthma/chronic bronchitis.

Abbreviations: EURONET-SAMM, European Network on Severe Acute Maternal Morbidity; SAMM, severe acute maternal morbidity; SMM, severe maternal morbidity; US-CDC, United States Centers for Disease Control and Prevention

^a^Excluding hospital length of stay
